# Patients’ health literacy and health behaviour assessment in primary health care: evidence from a cross-sectional survey

**DOI:** 10.1186/s12875-022-01809-5

**Published:** 2022-09-05

**Authors:** Kristina Šulinskaitė, Daiva Zagurskienė, Aurelija Blaževičienė

**Affiliations:** Department of Nursing, Lithuanian University of Health Sciences, Kaunas, Lithuania

**Keywords:** Health care, Primary care, Health behaviour, Health literacy, Patients

## Abstract

**Background:**

Health literacy is defined as a person's ability to find, understand, and use health-related information when making health-related decisions. Patients with lower health literacy more frequently face difficulties when they have health issues or need medical help. Such patients are less likely to visit health care facilities and receive less help, which subsequently leads to higher hospitalization and mortality rates. Patients with better health literacy skills pay more attention to their health behaviours.

**Methods:**

This is a cross-sectional survey conducted in two primary health care centres—one public and one private—in Lithuania. The study enrolled patients who were visiting family physicians (*n* = 399). The study used the Health Literacy Survey European Questionnaire (HLS-EU-Q47). Calculation of means and two independent samples were used for statistical analysis, and a correlation coefficient was calculated.

**Results:**

The majority (40.6%) of respondents had problematic health literacy, while only 7% had excellent health literacy. Better health literacy was observed among younger patients (aged below 30 years), residing in urban areas, having higher education, and living with a partner. Inadequate or problematic health literacy was noted among 83.6% of respondents aged 59 years and older; similar rates were also observed among patients with basic or primary education (76.1%), secondary education (76.6%), and divorced patients (86%). Respondents with better health literacy also had better health behaviours (*p* < 0.05).

**Conclusions:**

Health literacy is influenced by age, residence, education, and family status. Patients with better health literacy also reported better health behaviours.

## Background

Health literacy is the degree to which individuals have ability to find, understand, and use the basic health information and services needed to make appropriate health-related decisions. It is often associated with health outcomes and health system costs and influences the way in which communication is managed in the health care system [1, 2]. To promote health literacy among patients and improve their overall health, it is necessary for service providers to acquire skills related to health literacy and implement strategies, including evaluation of health literacy and appropriate interventions [2–4]. Health literacy basically refers to patients’ competences in changing their health care outcomes [5, 6].

Different levels of health literacy are directly related to self-empowerment—the patient's ability to independently understand, evaluate and use the provided information [7].

Low health literacy affects the quality of life as well as life expectancy and premature death [8, 9]. One-third of elderly people have problems reading and understanding health-related information, and poor understanding of such information is related to higher morbidity and mortality due to chronic diseases [10]. Low health literacy negatively affects patients' health, health-related behaviour, and the usage of health care resources [11]. A study in Australia showed that more than half of elderly people do not have sufficient health literacy skills for more complicated daily requirements [12].

Patients with higher level of health literacy can find health-related information independently. They also tend to understand the information provided by health care professionals more easily, are more likely to change lifestyle habits and more consciously try to sustain good health [7, 13].

Based on these findings, it can be presumed that new forms and measures need to be implemented in primary health care for the organization and management of services. Therefore, it is very important that primary health care professionals have robust knowledge about communication with patients, depending on their health literacy.

This study aimed to investigate patients' health literacy levels and assess the relationship between health literacy and health behaviour in primary care settings.

## Methods

### Study setting and population

The sample selection was based on the register of patients of the two primary health care settings in Kaunas city (public and private), Lithuania. In the study, patients older than 18 years were invited to participate in the study. The sample size (*n* = 399) was calculated assuming 95% significance and 5% error (the minimum sample for a total population of people in primary health care settings of the city was 382) [14]. The response rate was 97.3%.

### Study design

This is a cross-sectional survey conducted from October 2019 to January 2020.

### Instrument for measurement of health literacy

The study used the Health Literacy Survey European Questionnaire (HLS-EU-Q47), adapted in Lithuania. Previous studies and our study were shown that HLS-EU-Q47 had good internal consistency, reliability (Cronbach a > 0.90 for all items combined), and the interitem correlations were ≥ 0.40 (sufficient convergent validity).

Health literacy was assessed in three domains: health care (Items 1–16), disease prevention (Items 17–31), health promotion (Items 32–47) and in four stages of processing: access/obtain, understand, process/appraise and apply/use information relevant to health [15].

Literacy was measured using a 4-point Likert scale (4 = very easy; 3 = easy; 2 = difficult; 1 = very difficult; 0 = do not know). For the analyses, only responses 1–4 were included. The responses to all 47 items were transformed to a health literacy index ranging from 0 to 50:

$${\varvec{I}}{\varvec{n}}{\varvec{d}}{\varvec{e}}{\varvec{x}}=({\varvec{m}}{\varvec{e}}{\varvec{a}}{\varvec{n}}-1)\boldsymbol{ }\boldsymbol{*}50/3$$

Here ***index*** – health literacy index (total and domains);

***mean*** – items mean for every subject.

Higher index scores indicate better health literacy. The scores were then categorized based on the HLS-EU Consortium methodology into four groups [7]: below 26 points – inadequate, 26–33 points – problematic, 34–42 points – sufficient, and above 42 points – excellent [15, 16].

Likewise, an index for health behaviour literacy was calculated (Items 13, 14, 15, 16, 28, 29, 30, 31, 44, 45, 46, and 47). It was based on the same formula above.

### Statistical analyses

To compare the results of the survey in different groups of respondents according to sociodemographic characteristics, we combined groups of respondents with a relatively small number of respondents with others in the following order:

1. We combined one-yard and rural respondents into one group.
2. Respondents with primary, basic, and basic professional qualifications are combined into one group with secondary, secondary with professional qualifications and special—secondary education respondents— to another separate group.
3. We did not include the respondent who indicated the answer option “Other” in the answer about the session in the comparisons of results by session.

 

Additionally, to compare the results of the study in groups of respondents of different ages, all respondents by median age were 25 percent and 75 percent. We divide the quartiles into four groups:

1. Age up to 30 years.
2. Age of 31–47 years.
3. Age of 48–58 years.
4. Age of 59 years and older.

By following the steps above, we sorted the data and compared the results in different groups of respondents according to their sociodemographic characteristics [15].

The collected data were analysed and classified according to individual research objectives, from more general topics to more specific ones. Statistical data analysis was performed using "SPSS 24.0" (Statistical Package for the Social Sciences) statistical package [17]. Descriptive data statistics – absolute (n) and percentage frequencies (percent) – were used to assess the distribution of the analysed characteristics in the selected sample. For continuous variables, the means and standard deviations were calculated. Comparison of means in subgroups was conducted using Student’s t test for two independent samples and ANOVA (for three or more independent groups). Associations between health literacy indices and health behaviours were assessed using Pearson correlation. The significance level was set at *p* < 0.05.

## Results

### Study characteristics

The sample characteristics are presented in Table 1. We interviewed a total of *n* = 399 patients attending two primary health care centres – public and private. The mean age of respondents was 48 ± 19.4 years. The majority of respondents were women (57.6%), urban residents (78.4%), individuals with general secondary (36.6%) or higher education (37.8%), and individuals who were employed. According to marital status, the majority of respondents (55.9%) were married or living with a spouse (Table 1).

**Table 1:** Participant characteristics (*N* = 399)

Attribute		Respondents	
		**Abstract number**	**Percentage**
**Gender**	Men	169	42.4
	Women	230	57.6
**Age**	≤ 30 years	96	24.1
	31–47 years	101	25.3
	48–58 years	89	22.3
	≥ years	113	28.3
**Residence**	Countryside	86	21.6
	City	313	78.4
**Education**	Primary	5	1.3
	Lower Secondary	41	10.3
	General Secondary	146	36.6
	Junior College	56	14.0
	Higher	151	37.8
**Social status**	Employed	270	67.7
	Unemployed	21	5.3
	Student	24	6.0
	Retiree	83	20.8
	Other	1	0.3
**Family status**	Married or living with a partner	223	55.9
	Single	94	23.6
	Divorced	43	10.8
	Widowed	39	9.8

### Patients’ health literacy

**Fig. 1:**
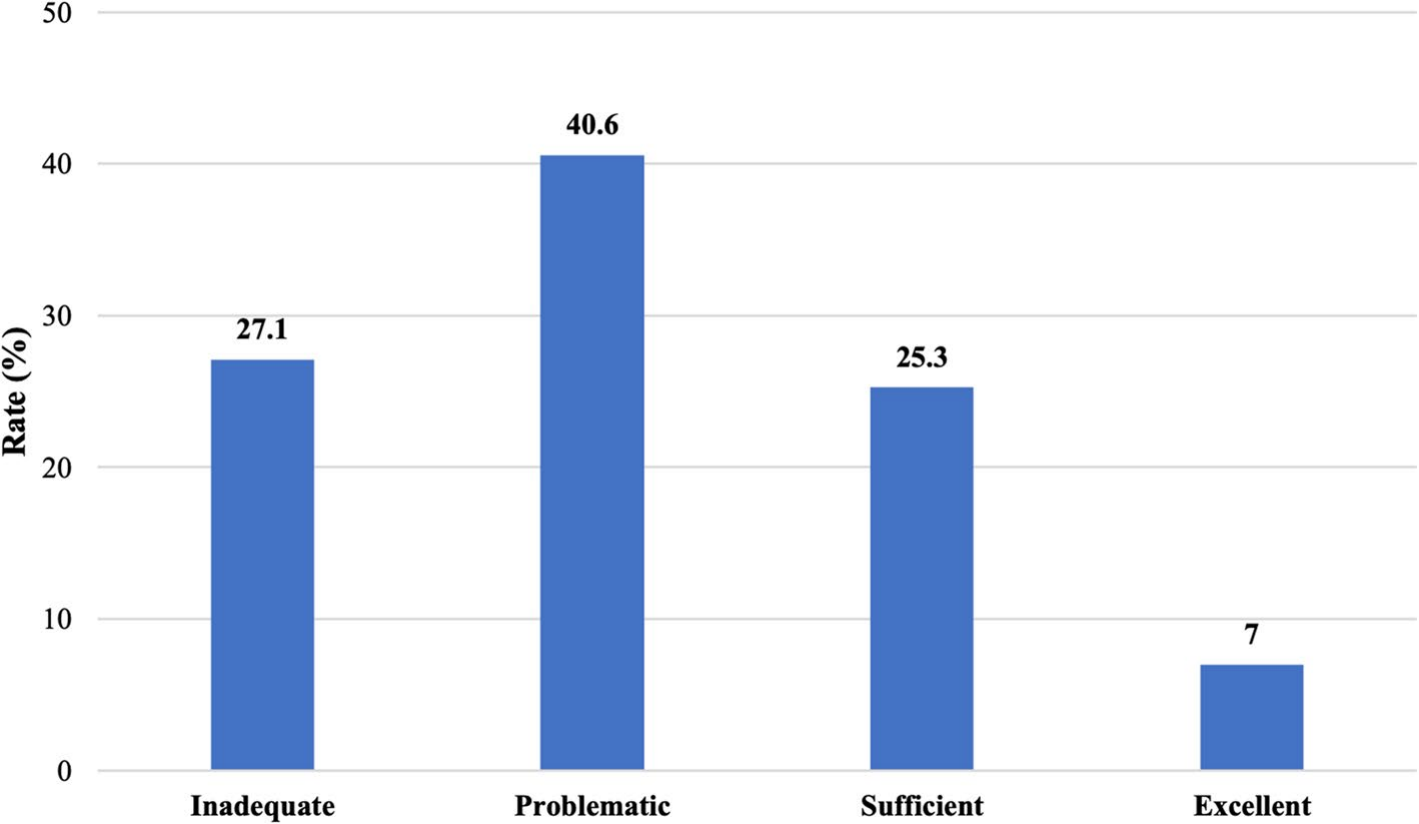
Patients’ health literacy

The study results showed that general health literacy among the majority of respondents was problematic, one quarter was inadequate, and slightly less was sufficient, while excellent literacy was observed only among 7% of respondents (Fig. 1).

**Fig. 2:**
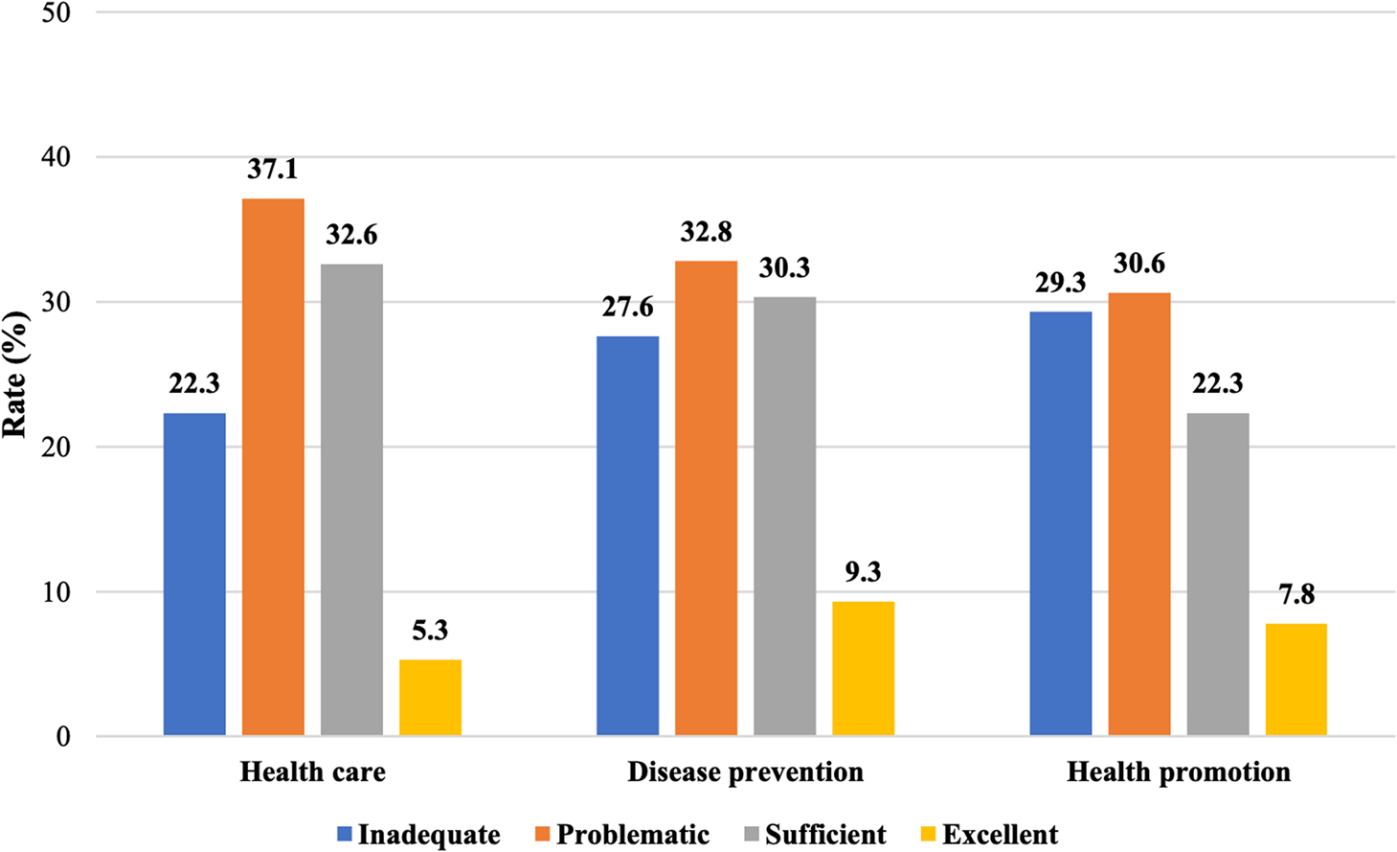
Patients’ health literacy in health care, disease prevention and health promotion indices

Analysis of health literacy by domains revealed the same pattern: in the health care domain, patients were mostly problematic (37.1%) and the smallest part was excellent (5.3%); in the disease prevention domain, they were 32.8% and 9.3%, respectively; and in the health promotion domain, they were 30.6% and 7.8%, respectively. Thus, it can be concluded that the largest proportion of patients has problematic health literacy, and the smallest proportion of patients has excellent health literacy (Fig. 2).

### Sociodemographic factors and health literacy

It was found that health literacy is associated with sociodemographic indicators. Sufficient and excellent health literacy was more common in patients aged below 30 years, among students, urban residents, persons with higher education and among people living with a spouse or a partner. The problematic and inadequate level of health literacy was more prevalent among patients aged above 48 years, countryside residents, and those with primary, basic, or secondary education, among unemployed, retired, divorced, or widowed subjects. However, sex did not play a significant role (*p* > 0.05) (Table 2).

**Table 2:** Relationship between respondents’ characteristics and health literacy levels

**Respondents’ characteristics**	**Health literacy (%)**				X^2^	*df*	*p*
	Inadequate	Problematic	Sufficient	Excellent			
**Age (years)**							
30	12.3	31.1	43.4	13.2	63.42	9	** < *0.001***
31–47	21.1	44.2	29.5	5.3			
48–58	29.0	51.0	16.0	4.0			
≥ 59	46.9	36.7	11.2	5.1			
**Residence**							
Countryside	38.4	38.4	14.0	9.3	29.69	9	** < *0.001***
Small town	12.2	40.8	40.8	6.1			
City	29.7	42.0	23.1	5.2			
Big city	11.5	38.5	38.5	11.5			
**Education**							
Primary, Lower secondary, General secondary with a professional qualification	43.5	32.6	19.6	4.3	29.95	9	** < *0.001***
Basic, Basic with a professional qualification	35.9	40.7	20.0	3.4			
Junior college	23.2	44.6	25.0	7.1			
Higher	15.2	41.1	32.5	11.3			
**Social status**							
Employed	21.1	44.1	27.8	7.0	52.58	9	** < *0.001***
Unemployed	28.6	38.1	23.8	9.5			
Student	8.3	20.8	58.3	12.5			
Retiree	51.8	36.1	7.2	4.8			
**Family status**							
Married or living with a partner	26.5	43.0	23.3	7.2	32.55	9	** < *0.001***
Single	14.9	34.0	40.4	10.6			
Divorced	39.5	46.5	11.6	2.3			
Widowed	46.2	35.9	15.4	2.6			

### Health literacy and positive health behaviour towards health care and disease prevention

Research shows that health literacy is associated with a healthier lifestyle and behaviour, so we wanted to analyse this aspect. Pearson correlation results demonstrated that the index of health behaviour significantly correlates with all indices of health literacy (*p* < 0.05). The strongest relationship was observed concerning total health literacy (*r* = 0.866), while the weakest relationship was observed in the health care domain of literacy (*r* = 0.761).

ANOVA also revealed significant associations (*p* < 0.05). It was found that higher health literacy is related to better health behaviours. This was observed not only with total health literacy but also with domain indices; patients with a higher level of health literacy were more likely to report better health behaviours (Table 3).

**Table 3:** Distributions of Health literacy and health behaviour

Health Literacy		Health behaviour				
**Areas**	**Levels**	***N***	**Vid**	**SN**	***F***	***p***
General	Inadequate	172	23.93	5.11	265.967	** < *0.001***
	Problematic	102	29.87	4.21		
	Sufficient	93	36.69	3.67		
	Excellent	32	43.29	4.01		
Health care	Inadequate	172	25.83	6.46	126.111	
	Problematic	102	30.81	5.47		
	Sufficient	93	36.97	4.35		
	Excellent	32	42.44	4.90		
Disease prevention	Inadequate	172	23.90	6.33	154.737	
	Problematic	102	30.74	6.86		
	Sufficient	93	37.00	4.55		
	Excellent	32	43.47	5.06		
Health promotion	Inadequate	172	22.07	5.79	246.551	
	Problematic	102	28.09	4.63		
	Sufficient	93	36.15	5.17		
	Excellent	32	44.02	3.89		

Summing up the results, it can be concluded that patients with better health literacy also report better health behaviours. Respondents with excellent or sufficient health literacy level had better health behaviours than those with problematic or inadequate health literacy level.

## Discussion

Improvement of health literacy may also improve a patient’s health condition, disease control and prevention [9]. Every country is making efforts to achieve high health literacy; however, the results are still not yet sufficient. Every third elderly person faces difficulties in searching, understanding, and applying health-related information in daily life [10]. Our study found that in general, health literacy among patients is mainly problematic, and only a small portion of them have excellent health literacy. One study in Germany also demonstrated that only 7.3% of respondents had excellent health literacy, while more than half of respondents had problematic literacy in health-related issues. People with problematic health literacy had difficulties searching, understanding, assessing, and applying health-related information [18]. Researchers in Denmark also supported these trends – based on general health literacy, the most common is the problematic level, and the least common is the excellent level of health literacy [19]. This suggests that among patients in European countries, low health literacy is predominant.

In our study, we found that low health literacy is related to untoward sociodemographic factors, such as lower education, absence of a life partner, older age, social status, and living in the countryside. Another study also found similar results; people with lower education were more likely to report tension with health-related issues when they needed to visit a health care setting and communicate with health care professionals. This shows that education has a significant effect on tension in health check-ups where people with better education face such issues less frequently [20]. It is noted that single patients, individuals with lower education, individuals and less income were less likely to seek and receive health care. Social inequalities manifest in lower health literacy among older adults, men, and adults from disadvantaged backgrounds, so they are most vulnerable due to skill reduction with ageing. Cognitive function and even a small deterioration of it with ageing have negative effects on health literacy. Therefore, innovative interventions are needed to reduce such problems of health literacy that emerge due to deteriorating health with ageing [20, 21].

Health literacy is associated with positive self-perception and better health behaviour. In our study, we found that patients with better health literacy also tended to report better health behaviour. A previous study in Lithuania conducted several years ago showed that 43.5% of respondents considered their health behaviour changes to be affected by health information. Younger respondents were more likely to change nutritional habits, middle-aged were more likely to increase physical activity, and 60 years and older were more likely to decrease salt consumption [22]. In 2016, researchers from Denmark reported that health literacy and the ability to understand health information are mediators between education and health behaviour. Patients with lower health literacy were less active in their health management [19]. Therefore, the analysis shows that health literacy, health indicators, and personal functioning are interrelated. Better health literacy is always associated with better cognitive function, fewer depressive symptoms, fewer chronic diseases, better daily mobility, and good physical condition [23].

The study results and previous research demonstrate the need for improvement of health literacy in all fields of health. Health literacy skills and lifestyle are among the main factors determining health. These aspects affect not only individuals but also public welfare; therefore, it is very important to consider how physicians, nurses, and other health professionals communicate health-related information, making it understandable, concise, and clear. Varying health literacy across populations increases the need for a health professional to adjust the provided information based on a person’s skills and expectations.

## Conclusions

The majority of the patients in primary health care had problematic or inadequate health literacy, which was associated with older age, urban residence, lower education, and single marital status. Respondents with higher levels of health literacy also reported better health behaviours. The results of the study show that health literacy is the most problematic in the areas of health care, disease prevention and health promotion.

Based on our research data and literature review we would like to provide some recommendations for improving health literacy in the community and society as well. At the state level, we would recommend more teaching hours in the curriculum about health literacy, to develop patient education programs and encourage health care providers to spend more time on patients’ health education.

At the municipal level (healthcare sector) we would suggest developing communication with the patient and his relatives about their health and how to protect it. To ensure easily accessible health-related information use more media and information technologies.

### Strengths and limitations

In Lithuania, studies on health literacy are insufficient, especially in the primary health care sector. Based on these findings, we demonstrated that health literacy in this country is similar to that in other European countries. The strength of this study is that it was one of the first studies of its kind that assesses patients' health literacy in primary health care, which accounts for 80% of health-related decisions.

Therefore, this study not only revealed the problem of health literacy but also suggested recommendations for health care professionals.

The limitation is that there was no opportunity to compare the literacy between the two settings because their number of visits and age structure were different.

## Data Availability

All data generated and analysed during this study are included in this published article.
